# Deep learning for histopathological segmentation of smooth muscle in the urinary bladder

**DOI:** 10.1186/s12911-023-02222-3

**Published:** 2023-07-15

**Authors:** Sridevi K. Subramanya, Rui Li, Ying Wang, Hiroshi Miyamoto, Feng Cui

**Affiliations:** 1 Thomas H. Gosnell School of Life Sciences, Rochester Institute of Technology, 1 Lomb Memorial Drive, Rochester, NY 14623 USA; 2 Golisano College of Computing and Information Sciences, Rochester Institute of Technology, 20 Lomb Memorial Drive, Rochester, NY 14623 USA; 3 Department of Pathology and Laboratory Medicine, University of Rochester Medical Center, 601 Elmwood Avenue, Rochester, NY 14642 USA

**Keywords:** Deep learning, Smooth muscle, Bladder cancer, Pathological slides, Segmentation

## Abstract

**Background:**

Histological assessment of smooth muscle is a critical step particularly in staging malignant tumors in various internal organs including  the urinary bladder. Nonetheless, manual segmentation and classification of muscular tissues by pathologists is often challenging. Therefore, a fully automated and reliable smooth muscle image segmentation system is in high demand.

**Methods:**

To characterize muscle fibers in the urinary bladder, including muscularis mucosa (MM) and muscularis propria (MP), we assessed 277 histological images from surgical specimens, using two well-known deep learning (DL) model groups, one including VGG16, ResNet18, SqueezeNet, and MobileNetV2, considered as a patch-based approach, and the other including U-Net, MA-Net, DeepLabv3 + , and FPN, considered as a pixel-based approach. All the trained models in both the groups were evaluated at pixel-level for their performance.

**Results:**

For segmenting MP and non-MP (including MM) regions, MobileNetV2, in the patch-based approach and U-Net, in the pixel-based approach outperformed their peers in the groups with mean Jaccard Index equal to 0.74 and 0.79, and mean Dice co-efficient equal to 0.82 and 0.88, respectively. We also demonstrated the strengths and weaknesses of the models in terms of speed and prediction accuracy.

**Conclusions:**

This work not only creates a benchmark for future development of tools for the histological segmentation of smooth muscle but also provides an effective DL-based diagnostic system for accurate pathological staging of bladder cancer.

**Supplementary Information:**

The online version contains supplementary material available at 10.1186/s12911-023-02222-3.

## Background

Smooth muscle is present in the wall of the hollow internal organs such as the blood vessel, gastrointestinal tract, and urinary bladder. The bladder wall is mainly composed of four layers, starting with the innermost epithelial lining called urothelium, lamina propria, muscularis propria (MP), and the outermost serosa or adventitia. Specifically, the lamina propria is the subepithelial layer which contains fibroblasts/myofibroblasts and capillaries/lymphatics, as well as muscularis mucosa (MM), fascicles of smooth muscle. Thus, the MM and MP are the two major types of smooth muscle fibers seen in the bladder. These fibers typically exhibit distinctive morphological features, often showing discontinuous, wispy, wavy fascicles in the MM vs. thick muscle bundles in the MP [1].

Bladder cancer, mostly urothelial carcinoma, is one of the commonly diagnosed malignancies worldwide [2]. There are clinically two distinct types of bladder cancer, non-muscle-invasive and muscle-invasive diseases. Remarkably, conservative therapy can be offered for non-muscle-invasive bladder cancer (NMIBC), including stage T1 disease showing tumor extension limited to the lamina propria where the MM is present, while more aggressive treatment options, such as systematic chemotherapy and radical cystectomy (RC), where the entire bladder is surgically removed, are often required for muscle-invasive bladder cancer (MIBC) with invasion into the MP. Thus, the distinction between MM invasion (T1/NMIBC) and MP invasion (MIBC) is critical in determining treatment plans. In particular, RC has a significant impact on the patient’s quality of life, leading to the need for a urinary diversion and permanent urinary ‘stoma’ over the abdomen.

Histopathological diagnosis of virtually all bladder cancers, including MIBC, is first made in the tissues biopsied/resected transurethrally. However, in transurethral resection (TUR) specimens stained with hematoxylin & eosin (H&E), it is often difficult to distinguish between the MM, which can be hyperplastic, and the MP, which can be partially destroyed or splayed by infiltrating cancer [3, 4]. To date, there are no biomarkers that are useful for objectively distinguishing the two types of muscle bundles in the bladder [5, 6]. It is thus often challenging for pathologists to differentiate between MM invasion and MP invasion in TUR specimens under microscope. Considering the critical differences in prognosis and treatment strategy between MM (NMIBC) and MP (MIBC) invasion, accurate detection of MM vs. MP muscle fibers in H&E-stained bladder tissues is of high clinical importance.

The enhanced use of digital pathology and advancement of modern machine learning (ML) and deep learning (DL) techniques in the field of medical image processing, has proven to improve identification and automation of tissue structures/abnormalities in histopathological images [7, 8]. These learning mechanisms can be categorized into supervised learning, weakly supervised learning, unsupervised learning, and transfer learning [9]. The supervised learning techniques are used to (1) identify objects (such as cells, glands, nuclei) [10] or make image-level predictions (such as cancer or non-cancer) with classification models [11]; (2) localize the objects with regression models [12]; or (3) delineate the contour of the objects with segmentation models [13]. The weakly supervised learning techniques exploit image-level annotations (such as cancer or non-cancer) to infer pixel/patch-level information [14]. That is, each histopathological image with cancer/non-cancer label forms a ‘bag’ and each patch/pixel extracted from that image is referred to as an ‘instance’. The goal of weakly supervised learning is to train a classifier with bag-level labels to predict both bag-level and instance-level labels. The unsupervised learning techniques aim to learn useful patterns of underlying data structure without the use of labels. Several unsupervised learning techniques are proposed including (1) autoencoders that were used for modeling the stochasticity [15] or disentangling visual features [16]; (2) Generative Adversarial Networks that were used for cell and tissue classification [17]; (3) self-supervised learning methods that were used to classify and segment histopathological images [18]. Lastly, transfer learning is typically done using ImageNet pretrained models such as GoogleNet, Inception-V3, MobileNet to detect breast cancer metastasis [19, 20] or predict Gleason score of prostate cancers [21].

Within the supervised learning techniques, two categories of classification models have been employed for digital pathology tasks such as cancer detection and classification, cancer staging, and survival prediction. Models in the first category focus on patch-based classification for disease prediction tasks with whole-slide-images (WSI) [22–26] using traditional convolutional neural network (CNN) architectures. However, these WSIs will be preprocessed and converted to patches of defined size and assigned to a particular class before model training is initiated. Further in the paper, we refer these architectures as patch-based models. A patch-based pipeline was developed to differentiate cancer and normal tissues using WSI from breast cancer tissues [27]. Models in the second category focus on pixel-wise semantic segmentation of the image into regions that belong to different classes [28–31]. As one of the most successful semantic segmentation models which we refer to as pixel-based models, U-Net has been applied to detect tumors in the lungs and brain [32]. In this category, pixel-based CNNs are usually applied on an image, where each pixel of the image is assigned to a particular class. Although both patch-based and pixel-based approaches are successful in classifying and segmenting medical images at different scales, direct comparison between them is rare. A recent study [33] used patch-based (AlexNet) and pixel-based (U-Net) models to identify and classify mitosis in histopathological WSI, as a quantitative measure in breast cancer diagnosis. The authors used only one metric, accuracy, to compare both models. The AlexNet model was found to achieve 95% accuracy whereas the U-Net reached 99% accuracy. However, a thorough comparison between both model categories requires: (1) more models from each group; and (2) more evaluation metrics.

In this study, we applied well-known DL models (patch-based and pixel-based approaches) to accurately differentiate MP from all non-MP tissues, including MM, in H&E-stained bladder specimens. For the patch-based approach, patches were extracted such that each patch was labeled as either MP or non-MP. Several state-of-the-art patch-based models were used including VGG16 [22], ResNet18 [23], SqueezeNet [24], and MobileNetV2 [25, 26], which were pre-trained by ImageNet data. By contrast, for the pixel-based approach, every original image and the corresponding mask image were divided into an equal number of image patches and mask patches, with each pixel in the mask patch representing the label (MP or non-MP) of the corresponding pixel in the image patch. The state-of-the-art pixel-based models chosen for our analysis were U-Net [32], MA-Net [34], DeepLabv3 + [35], and FPN [36].

Our contributions are summarized as follows:

- This is the first computational analysis of morphological features of smooth muscle in H&E-stained images. We apply recent and well-known DL-based models to the data and create a benchmark for further research using current or related datasets.
- We perform an extensive comparison on speed and performance between patch-based models and pixel-based models in segmenting smooth muscle tissues. Pros and cons of both approaches are discussed.
- We present an efficient computational framework to automatically pre-process smooth muscle images of bladder, which are used as an input to a suite of DL-based models. Segmented images with highlighted MP regions are produced as an output.
- We create an end-to-end DL-based diagnostic system that can be used in clinical applications on staging bladder cancers to reduce pathologists' time and effort.

The remainder of this paper is organized as follows. Section 2 explains the details of proposed methodology including hardware specification and model architectures, as well as model training and inference methods. Experimental results that validate the proposed methodology are presented in Section 3 and discussion of our findings and relevant studies are given in Section 4.

## Methods

### Software and hardware

The proposed methodology was executed on a workstation with hardware and software specifications as described in Supplementary Table 1. The workstation had 16 GB of RAM, 6 GB of graphical memory (GPU), i7 6 core processor, and Windows 10 operating system. All programming tasks were performed in Spyder, an integrated development environment from Anaconda.org, except the Ground Truth preparation, for which MATLAB R2020b Image Labeler tool was used. In Spyder, all DL analyses were accomplished using PyTorch [37], a Python-based scientific computing package that included functionality to use the power of system GPUs, thereby utilizing available resources and leading to time-efficient analysis. PyTorch also incorporated automatic efficient differentiation libraries that were useful for implementing DL neural networks. For all the numerical computations NumPy library [38] was used. For image reading/manipulations and plots, OpenCV and Matplotlib were utilized, respectively. For file operations such as reading the file and writing results to a file, Pandas [39] library was used.

### DL models

**Fig. 1:**
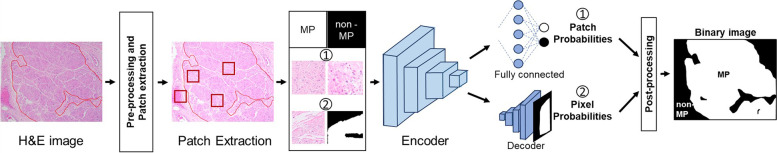
Summarized methodology. The annotated H & E-stained images are pre-processed and split into patches of defined size. These patches are extracted in two ways to create two datasets: ① patch-based: each patch is either fully MP or fully non-MP and ② pixel-based: each patch includes respective ground truth mask patch where white pixel corresponds MP and black pixel corresponds non-MP. Selected CNN models are trained on the patch-based dataset and deep learning models are trained on the pixel-based dataset. The trained models for patch-based approach and pixel-based approach will be used to semantically segment any given H&E-stained image into MP and non-MP regions

The proposed methodology is summarized in Fig. 1. To semantically segment the H&E-stained tissues into MP and non-MP regions, we applied two types of DL-based approaches: patch-based approach and pixel-based approach. A detailed explanation of the above-mentioned approaches is described in the following subsections.

### Patch-based approach

**Fig. 2:**
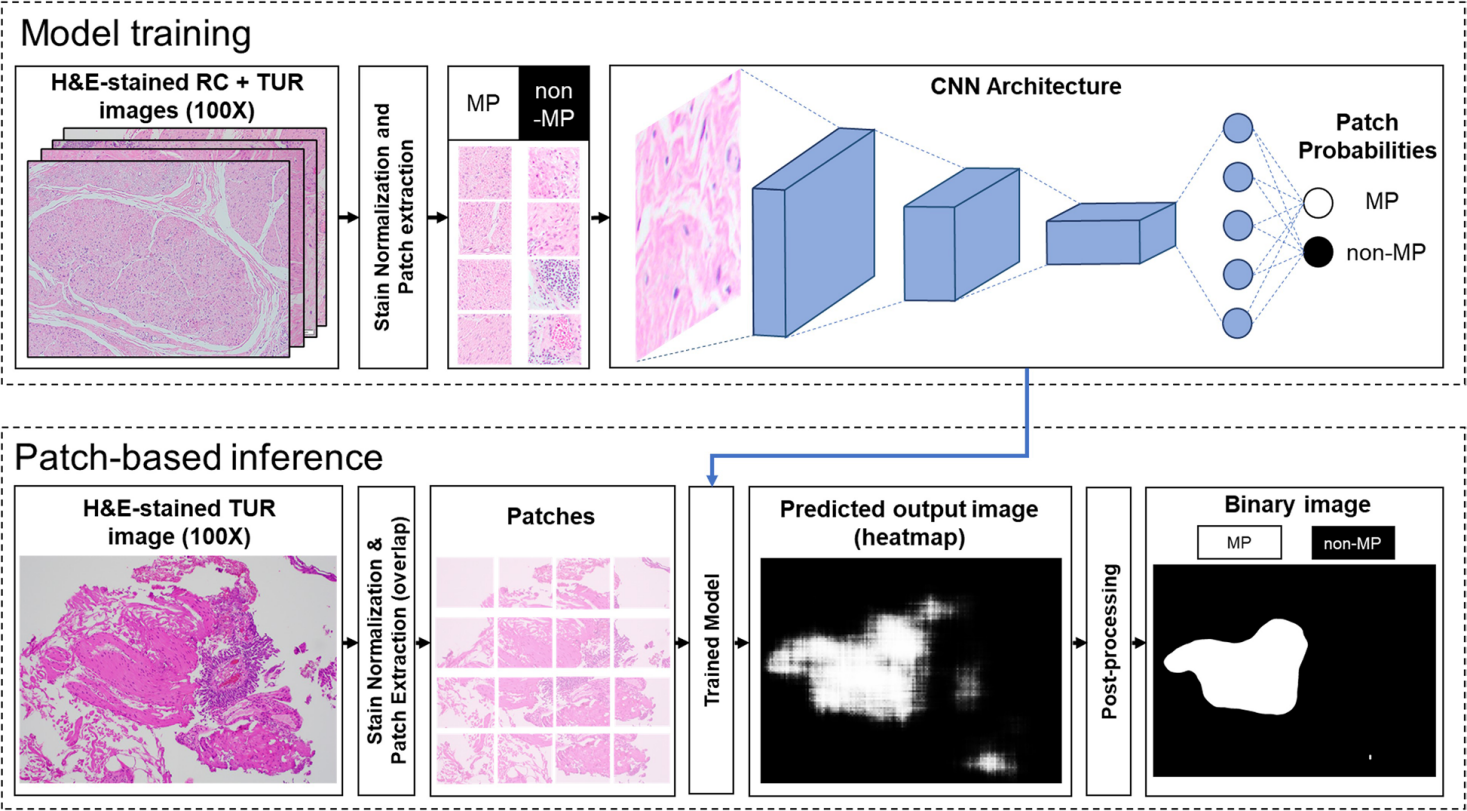
Flow diagram of the patch-based approach for classification and semantic segmentation of MP and non-MP regions from bladder H&E-stained histopathological images. In the model training step, the annotated images are stain normalized and split into patches with labels as either MP or non-MP. The CNN architecture is trained on that dataset to classify each patch as MP or non-MP region. In the patch-based inference step, the test image is stain normalized and split into patches. Each patch is passed through the trained CNN architecture and the patch probability is assigned to the center pixel of the patch. All the patch probabilities are merged together to form a segmented test image. Thus, the predicted output image contains either white pixels (MP) or black pixels (non-MP). This output image is further post-processed to obtain a binary image with smooth edges and minimal noisy pixels

Four state-of-the-art CNN models were used for analysis, including VGG16, ResNet18, SqueezeNet, and MobileNetV2 (see Table 1 for their architectures). Figure 2 shows an overview of the analysis that consists of two steps: model training and model inference. In the model training step, the images from the training dataset were passed through stain normalization technique and were divided into non-overlapping patches. The patches were extracted such that each patch included either MP-only or non-MP-only region and was labelled as MP or non-MP, respectively. Since each patch was a fully MP or non-MP patch, some parts of the original images that contained both MP and non-MP areas were unused. Next, the dataset consisting of patches and their corresponding labels (MP v/s non-MP) was passed through the aforementioned models that performed binary image classification. For all these models, instead of using random weight initialization, we chose to use the pre-trained weights from the ImageNet dataset [40] through transfer learning [41], a common practice in ML or DL research to improve image classification performance [27, 42]. Since the ImageNet dataset comprised natural images and did not contain any medical-related images, we chose to retrain all the layers of the whole network and updated the pre-trained weights of all models except VGG16, where only the weights in the last layer of the VGG16 model was updated as it contained a large number of parameters. Also, in each of the four architectures, we changed the last layer (classifier layer) to accommodate for two-class image classification, in our case (i.e., MP or non-MP). The trained models were then used to perform patch-based inference as explained below.

**Table 1:** Architecture of patch-based and pixel-based models

Patch-based models				Pixel-based models			
**Network**	**Depth (layers)**	**Trainable parameters (× 10**^**6**^**)**	**Training time (hours)**	**Network**	**Encoder**	**Trainable parameters (× 10**^**6**^**)**	**Training time (hours)**
VGG16	16	119.55	11.16	U-Net	ResNet18	14.33	14.10
ResNet18	18	11.18	4.72	MA-Net		21.68	16.47
SqueezeNet	18	0.74	4.40	DeepLabv3 +		12.33	12.58
MobileNetV2	53	2.23	6.19	FPN		13.05	11.74

In the inference step, our aim was to semantically segment the test images into MP and non-MP regions and thus, we assessed test images individually. First each image was stain normalized and divided into patches that overlapped each other. The reason was that when a patch was passed through the trained model, we obtained a probability of that patch being MP. To correctly account for transition between MP and non-MP regions, we empirically decided that patches should be 96% overlap (in other words we used a sliding window of size 10 pixels). Patches were passed through the trained models one by one and the patch probability value of being MP was assigned to the central pixel of the patch, which finally resulted in a small-sized heatmap representation of the predicted output image with values ranging from 0 to 1. This probability heatmap image was interpolated to the same size as that of the input image using nearest-neighbour interpolation. To reduce the effect of artifacts (caused by upsampling), we used a simple averaging filter (low-pass filter) as a part of post-processing. Each value in the heatmap image corresponds to the probability that a fixed-size patch is predicted to be MP. To convert the probability heatmap to a binary image representation, an optimal threshold was needed. As such, the threshold was determined as shown below using adaptive thresholding,

1 $${Y}_{t}=True\ positive\ rate-False\ positive\ rate$$

2 $$\widehat{t}=Y , Y\in {R}^{T}, T = number\ of\ thresholds$$

where ${Y}_{t}$ represented the Youden’s J statistic [43] defined as the difference between the true positive (TP) rate (Sensitivity) and false-positive (FP) rate (i.e., 1—Specificity). The TP rate and the FP rate were determined by comparing the probability heat map against the binary ground truth at the pixel-level. A threshold $\widehat{t}$ was determined such that it maximized the Youden’s J statistic. The probability heatmap representation was converted to binary image representation using this threshold $\widehat{t}$, where each pixel was now labelled as either MP (pixel value: 255) or non-MP (pixel value: 0). Lastly, we processed the binary image to remove the isolated noisy pixels (i.e., single/few positive pixels in the midst of negative pixels or vice versa, present due to false predictions from trained models) and high frequency artifacts. Hence, we used two types of filters. First, we used a median blur filter (kernel size = 155 × 155 pixels) which reduced the noise effectively. Next, we used a simple averaging filter (kernel size = 25 × 25 pixels) to smoothen the boundary pixels and applied Otsu thresholding which finally resulted in a smoothed binary image representation. The post processing steps to obtain the final output image is shown in Supplementary Figure 1. The same procedure was used to semantically segment all images into MP and non-MP regions in the test dataset.

### Pixel-based approach

**Fig. 3:**
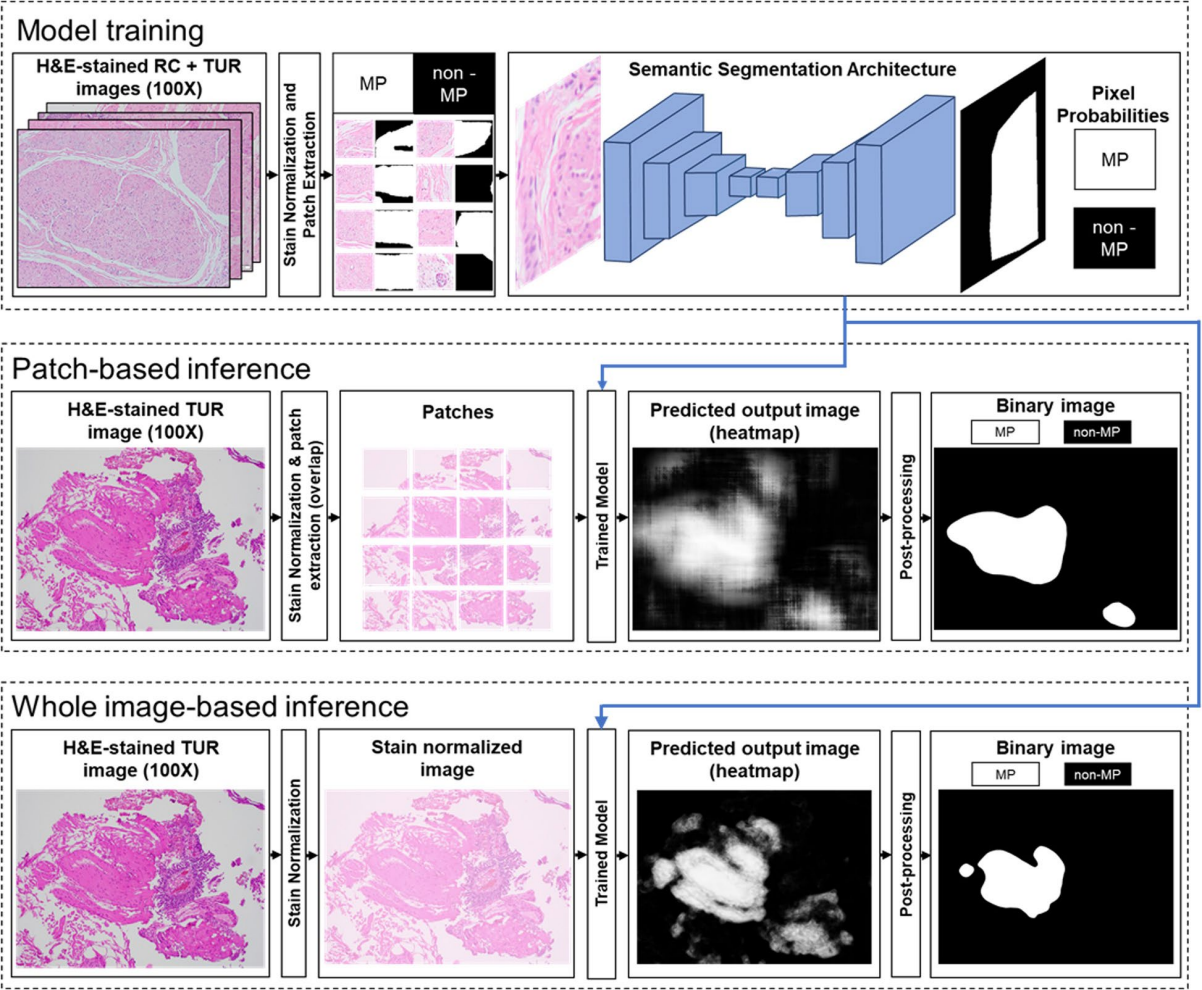
Flow diagram of the pixel-based approach for classification and semantic segmentation of MP and non-MP regions from bladder H&E-stained histopathological images. In the model training step, the annotated images are stain normalized. The stain normalized images and their corresponding ground truth masks are split into patches. The semantic segmentation architecture is trained on that dataset to classify each pixel as MP or non-MP region. In the patch-based inference step, the test image is stain normalized and split into patches. Each patch is passed through the trained semantic segmentation architecture to obtain a similar patch where each pixel of the patch is assigned a probability for MP and non-MP. All the predicted patches are merged together to form a segmented test image. Thus, the predicted output image contains either white pixels (MP) or black pixels (non-MP). This output image is further post-processed to obtain a binary image with smooth edges and minimal noisy pixels. In the whole image-based inference step, the test image is stain normalized and directly passed through the trained semantic segmentation architecture to obtain a segmented test image. The predicted output image contains either white pixels (MP) or black pixels (non-MP). The output image is post-processed similar to the patch-based inference step

For the pixel-based methods, we used state-of-the-art semantic segmentation models such as U-Net, MA-Net, DeepLabv3 + , and FPN (see Table 1 for their architectures). ResNet18 was chosen as an encoder to compare the performance of these four models. Figure 3 shows an overview of the analysis that consists of two steps: model training and model inference. In the model training step, all training images were first stain-normalized. These images and their corresponding binary ground truth images were divided into non-overlapping patches. However, unlike the patch-based approach, the patches were extracted such that each patch included either MP or non-MP region or both. The reason was that each pixel of the patch was labelled as either MP or non-MP. Thus, each extracted patch had an equal-sized binary ground truth mask. As a result, the entire image dataset could be effectively utilized for training the four semantic segmentation models listed above. The input patches and the corresponding binary ground truth masks were fed into the models to perform pixel-wise binary classification.

To assess the trained model performance, we used two independent inference methods: patch inference and whole image inference. The working of the patch inference was similar to the patch-based approach described above. However, as we obtained the MP probability for each pixel in a patch, we arranged these probability patches to obtain a heatmap representation of the predicted output image with size equal to that of the input image. By contrast, for whole image inference, test images were directly fed into the trained model without dividing the images into patches. The output image obtained was a probability heatmap whose size was the same as that of the input image and each pixel value indicated the probability that the pixel belonged to the MP class. Next, the optimal threshold was determined to convert the predicted probability heatmap image to an output image as the value that maximized the Youden’s J statistic, as shown in Eqs. (1) and (2). The obtained binary image was passed through the median blur (kernel size = 155 × 155 pixels), averaging filter (kernel size = 25 × 25 pixels) and Otsu thresholding to obtain a smooth binary image, as shown in Supplementary Figure 1. The methods used in patch and whole image inference were applied individually to all the images of the test dataset to semantically segment each image into MP and non-MP regions.

### Hyperparameter selection

To determine optimal model hyperparameters (Table 2), we employed trial-and-error experimentation using a validation dataset. For the patch-based approach, we used 30 epochs and chose a batch size of 32 [44]. The learning rate was chosen to be 0.001 and the stochastic gradient descent (SGD) optimization algorithm was used to update the weights. In addition to the static learning rate, we experimented with a decaying learning rate and found that with a decaying learning rate, the training time was increased by a few hours and we got either the same results as with static learning rate or less than that (data not shown). Therefore, we decided to continue with a static learning rate. We used the cross-entropy loss function, which was generally used in image classification tasks. For the pixel-based approach, number of epochs were empirically chosen to be 30 epochs. Smaller batch sizes are commonly used for semantic segmentation tasks, and thus, we used a batch size of 4 in our analysis. The cross-entropy loss function was used to determine the loss between predicted and ground truth masks. To optimize the loss function and update the weights we used a learning rate of 0.0001 with Adam optimizer. For both the approaches, we used weighted cross-entropy loss function to ensure unbiased model training as our dataset contained more negative (non-MP) classes compared to positive (MP) classes and weighted random subsampling to make sure that each batch of defined size encounters a proportional number of MP and non-MP classes. The class weights for weighted cross entropy loss function and weighted random subsampling were calculated as reciprocal of the number of patches/pixels belonging to a particular class respectively for each approach.

**Table 2:** Hyper-parameters of patch-based and pixel-based models

Hyper-parameters	Patch-based models	Pixel-based models
Batch size	32	4
Epochs	30	50
Optimization algorithm	Stochastic Gradient Descent	Adam (beta1 = 0.9, beta2 = 0.999)
Learning rate	0.001	0.0001
Criterion/ Loss function	Cross-Entropy	Cross-Entropy

### Evaluation metrics

As described in both patch-based and pixel-based approaches, the final output was the post-processed predicted binary image with MP and non-MP regions highlighted in different colors. To assess the model performance, commonly used pixel-level evaluation metrics were used, particularly for medical images, such as mean Jaccard index, mean Dice coefficient, pixel-wise accuracy, precision, recall, specificity, and F1 score. The basic components describing these metrics involved: TP representing the total number of MP pixels in ground truth correctly predicted as MP; true negative (TN) representing the total number of non-MP pixels in ground truth correctly predicted as non-MP; FP representing the total number of non-MP pixels in ground truth incorrectly predicted as MP; and false negative (FN) representing the total number of MP pixels in ground truth incorrectly predicted as non-MP.

Jaccard Index, also known as Intersection Over Union, is the ratio of the area of overlap to the area of union between the predicted image and the ground truth image. We determined the mean Jaccard Index by taking an average of class specific Jaccard Indices, each for MP and non-MP using the Eq. (3).

3 $$Jaccard\ Index=\frac{TP}{TP+FP+FN}$$

Dice coefficient is a statistical measure to determine the similarity between the predicted image and the ground truth image. It emphasizes only the positive class similarity and does not account for the negative class. Thus, we determined the full image Dice coefficient (MP and non-MP regions) by computing the average of class-specific Dice coefficients, each for MP and non-MP using Eq. (4).

4 $$Dice\ coefficient= \frac{2*TP}{2*TP+FP+FN}$$

Global pixel-wise accuracy indicates the fraction of correctly predicted pixels to the total number of pixels, and it is represented in Eq. (5).

5 $$Pixel\ Accuracy=\frac{TP+TN}{TP+TN+FP+FN}$$

The precision or the positive predicted value is the measure of correctness. It evaluates how “precisely” a model predicts a given pixel to the positive class, in our case, the MP class. The precision value was determined as shown in Eq. (6).

6 $$Precision= \frac{TP}{TP+FP}$$

Recall or sensitivity or the TP rate corresponds to the accuracy of positive cases. It was defined as the ratio of TPs to the total number of predicted positives, as represented in Eq. (7).

7 $$Recall= \frac{TP}{TP+FN}$$

Specificity or TN rate determines the non-MP class accuracy. As shown in Eq. (8), specificity was calculated as a ratio of total TNs to the total number of predicted negatives.

8 $$Specificity= \frac{TN}{TN+FP}$$

F1 score is a metric defined as the harmonic mean of precision and recall, as presented in Eq. (9). The higher value of the F1 score signifies how well the model predicts the positive class.

9 $$F1\ score= \frac{2*Precision*Recall}{(Precision+Recall)}$$

## Results

### Histopathological images

Upon approval from the Institutional Review Board at the University of Rochester Medical Center (URMC), a total of 303 images of H&E-stained bladder tissues from RC (8 patients—237 images of size 1920 × 1440 pixels) and TUR (8 patients—66 images of size 2448 × 1920 pixels) were collected from the Department of Pathology and Laboratory Medicine at URMC. The images were captured under 100X total magnification using an Olympus BX43 microscope attached with a high-resolution camera (DP27). In these images, MP and non-MP regions were manually segmented by board-certified pathologists (YW and HM). Twenty-six out of 303 images were excluded due to the presence of both MM and MP in the same image (*n* = 12), leading to no patch extraction, or difficulty for the pathologists in morphologically distinguishing between MM and MP (*n* = 14). As a result, 277 (= 303—26) images, including 214 from RC and 63 from TUR, were used to train and test the proposed models.

### Ground truth preparation and data pre-processing

The images annotated by the pathologists were used to prepare ground truth labels. The procedure used to generate labels for all histopathological images in our dataset is shown in Supplementary Figure 2. In short, a freehand drawing tool based on the GrabCut segmentation algorithm [45] in MATLAB was used to manually mark the region of interest. The output from the tool was a bi-level mask which was then converted to a binary image. The MP and non-MP tissues were represented as white and black regions corresponding to pixel values of 255 and 0, respectively.

**Fig. 4:**
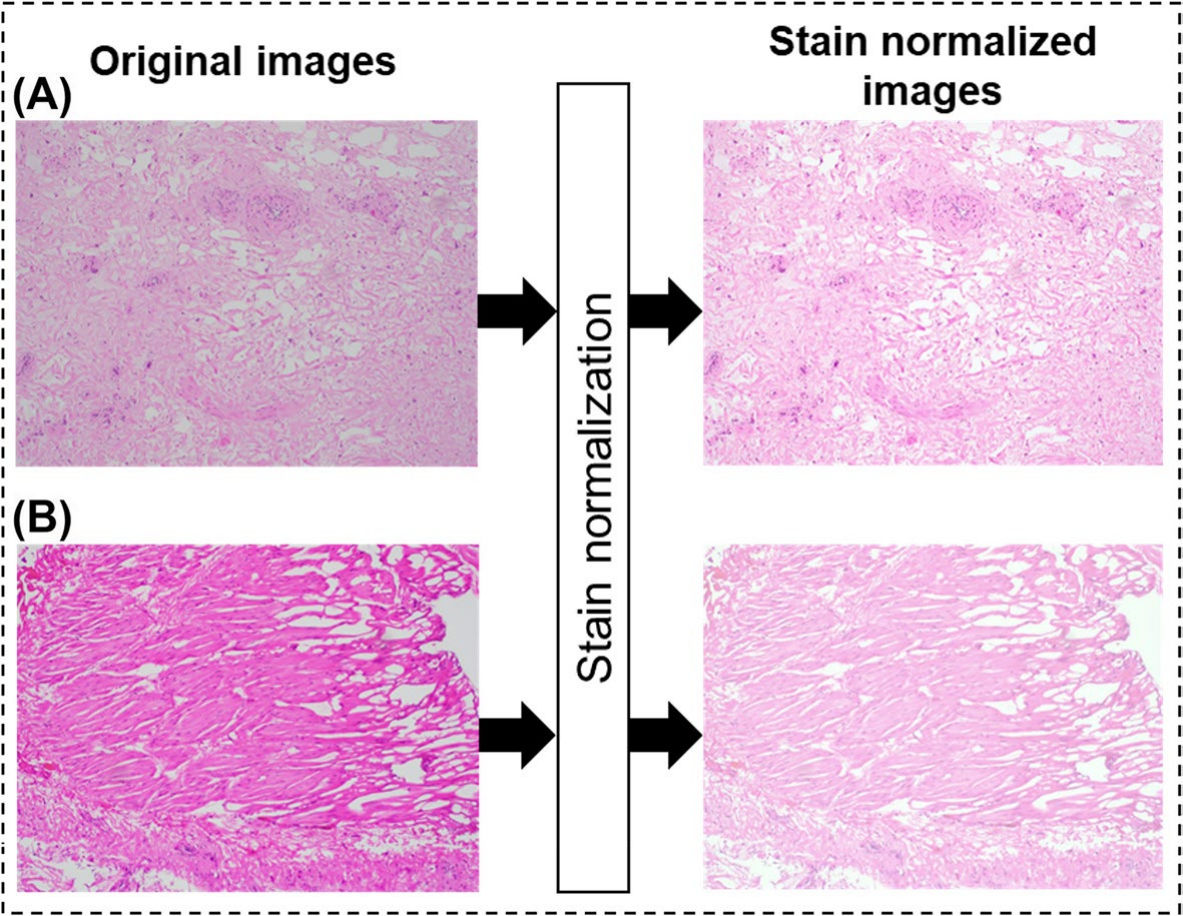
Effect of Reinhard stain normalization on H&E-stained histopathological images. Images are taken from radical cystectomy (**A**) and TUR specimens (**B**) at 100 × total magnification

All TUR images and their corresponding binary ground truth images were resized to 1920 × 1440 pixels using bilinear and nearest-neighbor interpolation, respectively, to maintain uniformity across all images. We observed considerable variability in the staining intensity among the images, especially between those from RC versus TUR. To alleviate these staining intensity differences, we applied Reinhard stain normalization [46], a standard color transferring technique that could impart the color of a chosen reference image to all. This normalization resulted in a dataset with uniform stain consistency among all images (Fig. 4). These stain-normalized images were used as input for our analysis. Since the proposed methods aimed to segment TUR images into MP and non-MP regions, we trained and tested models using ninefold cross validation technique, to ensure equal distribution of images in each fold. The 63 TUR images were divided into 9 sets, each with 7 images. In each fold, the model was trained using 214 RC images + 56 TUR images (except holdout set) and the holdout set (7 images) was tested using the respective trained model. The training images with dimensions of 1920 × 1440 pixels were divided into non-overlapping patches of 240 × 240 pixels. The patches were extracted in the size of 240 × 240 pixels because both the dimensions of the original image were divisible by the defined patch size, resulting in complete utilization of the input image without the need for truncation or padding. The training patches were further divided into 80% training set and 20% validation set using a stratified sampling technique [47], which ensured equal distribution of the classes in both training and validation datasets. The validation set was used as an indicator to prevent overfitting of the data during model training.

### Model training and inference of patch-based approach

For patch-based approach, the total number of patches extracted varied in each fold depending on the training images in a range between 8,718 and 8,813 patches. With predefined hyperparameters (Table 2), we trained all four CNN-based models using ninefold cross validation. The performance of four patch-based models was evaluated by a suite of metrics (Supplementary Table 2). We found that MobileNetV2 performed the best. Except for specificity, all the other metrics of MobileNetV2 were higher in comparison with those in other models. In addition, SqueezeNet provided stiff competition to MobileNetV2 with all evaluation metrics being very close to those of MobileNetV2. For ResNet18 and VGG16, their evaluation metrics were the least. Among different metrics, the mean Jaccard index and mean Dice coefficient were considered as best metrics to decide on the superiority of the model in the segmentation tasks. We observed that MobileNetV2/SqueezeNet had higher mean Jaccard (0.74/0.73) and Dice coefficient (0.85/0.84), compared to ResNet18/VGG16 (0.72/0.71 and 0.83/0.82, respectively).

**Fig. 5:**
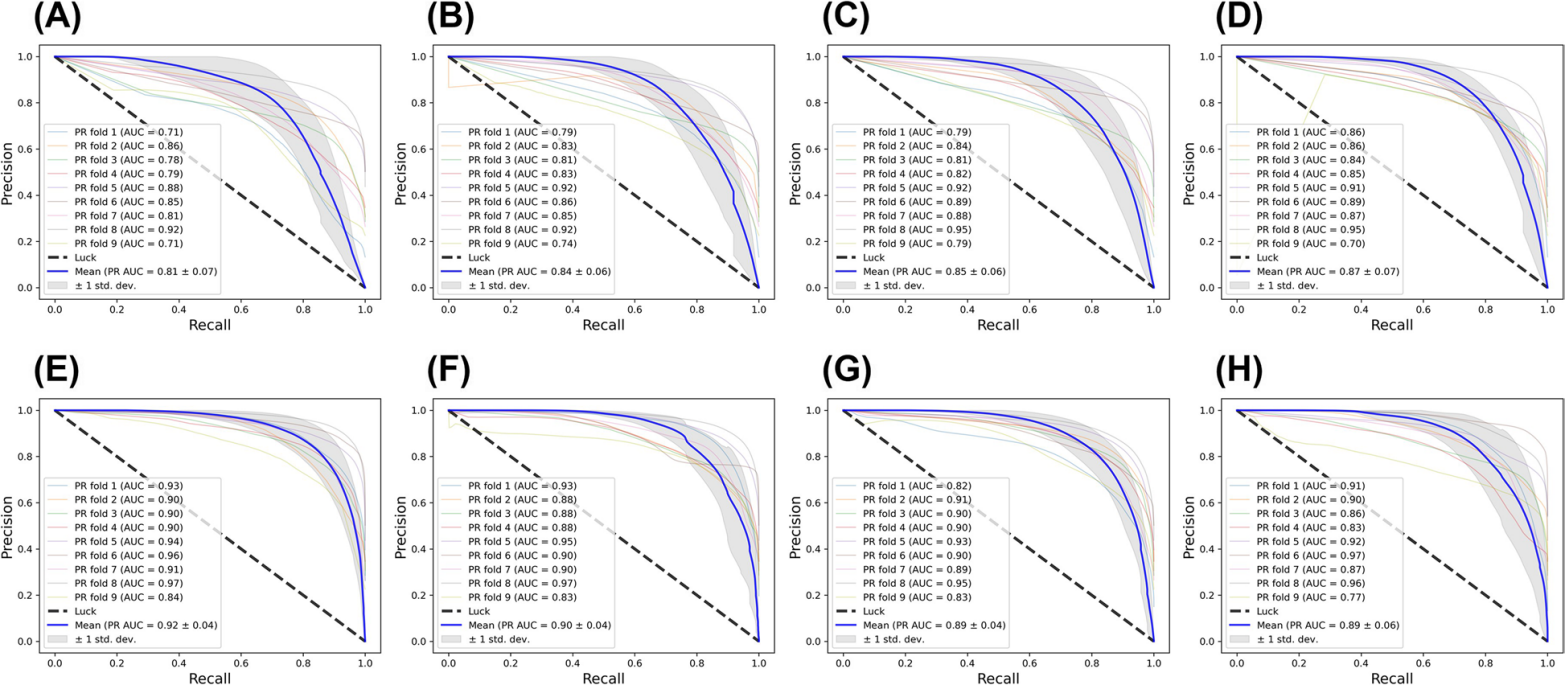
Precision-Recall (PR) curve for classification of MP and non-MP regions by VGG16 (**A**), ResNet18 (**B**), SqueezeNet (**C**), MobileNetV2 (**D**), U-Net (**E**), MA-Net (**F**), DeepLabv3 + (**G**) and FPN (**H**). Both patch-based models (**A**-**D**) and pixel-based models (**E**–**H**) were trained and tested using ninefold cross-validation. Seven TUR images were evaluated for each fold to calculate PR curves and corresponding PR-AUC values are indicated in the caption. The mean PR curve (blue) and standard deviation (grey shaded region) are provided for the models

**Fig. 6:**
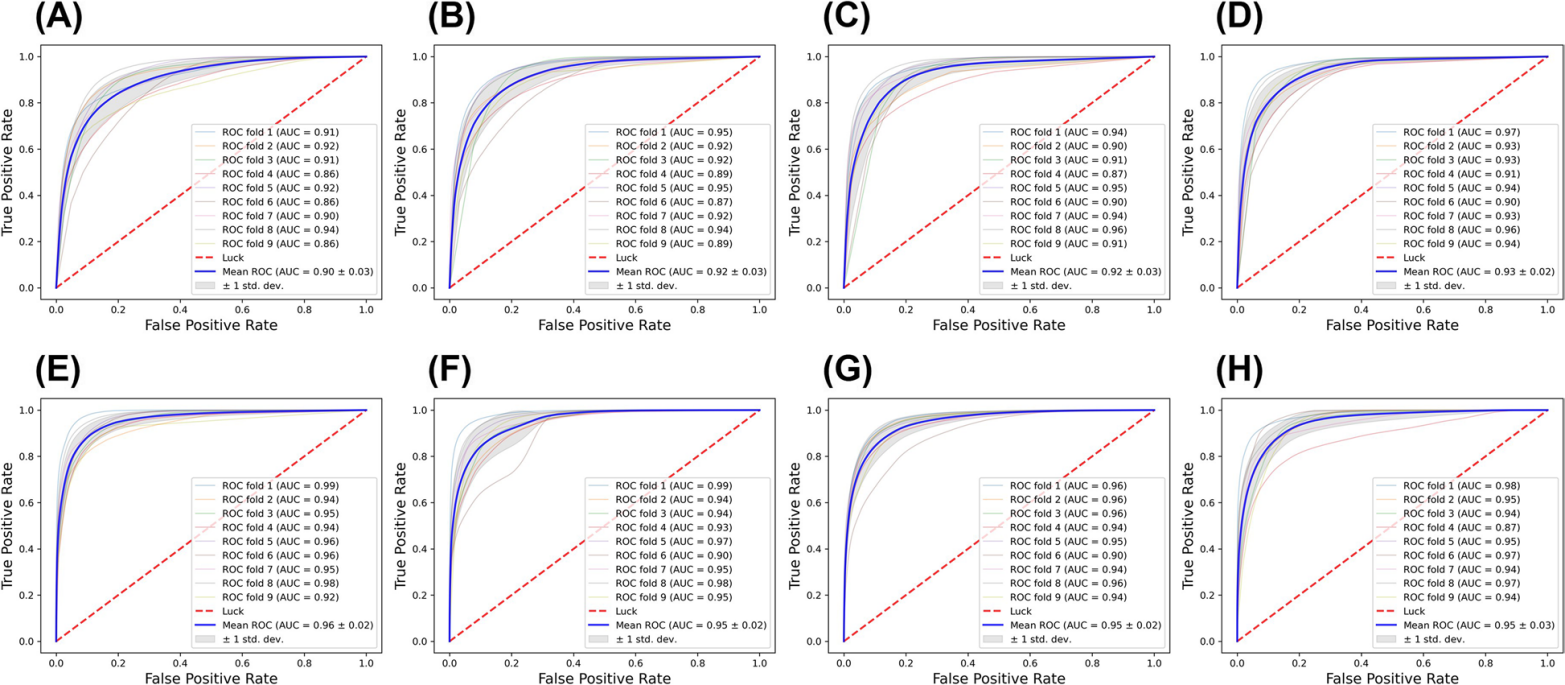
Receiver Operating Characteristic (ROC) curve for VGG16 (**A**), ResNet18 (**B**), SqueezeNet (**C**), MobileNetV2 (**D**), U-Net (**E**), MA-Net (**F**), DeepLabv3 + (**G**) and FPN (**H**). Both patch-to-label models (**A**-**D**) and pixel-to-label models (**E**–**H**) were trained and tested using ninefold cross-validation. Seven TUR images were evaluated for each fold to calculate ROC curves and corresponding ROC-AUC are indicated in the caption. The mean ROC curve (blue) and standard deviation (grey shaded region) are provided for the models

Precision-Recall (PR) curves (Fig. 5A-D) and Receiver Operator Characteristic (ROC) curves (Fig. 6A-D) were plotted for all the models. The PR Area under Curve (AUC) scores and ROC AUC scores were provided in the plots. Consistently, the MobileNetV2/SqueezeNet models had high mean PR AUC (0.87/0.85) and mean ROC AUC (0.93/0.92) values in comparison with the ResNet18/VGG16 models showing mean PR AUC of 0.84/0.81 and mean ROC AUC of 0.92/0.90. Hence, based on the evaluation metrics, we concluded that the MobileNetV2 and SqueezeNet models were the best performing patch-based models, with ResNet18 and VGG16 models providing stiff competition.

### Model training and inference of the pixel-based approach

For pixel-based models, the total number of patches extracted in each fold were 12,960 patches, which were higher than patches extracted for the patch-based approach. This was because each pixel of a patch was labelled and a patch could contain pixels with different labels (see Methods). With predefined hyperparameters (Table 2), we trained all four pixel-based models using ninefold cross validation. The model performance was evaluated by two inference approaches: patch inference and whole image inference (see below).

#### Patch inference

Patch inference is an approach similar to that of the inference for the patch-based approach, where overlapping patches are extracted from test images. Supplementary Table 3 shows the evaluation metrics for all the pixel-based models. We found that the U-Net performed the best in all metrics. The U-Net model outperformed all the other models with the mean Jaccard index being 0.78. The next best performing models were MA-Net and DeepLabv3 + , where all metrics except the mean Jaccard index were equal to that of U-Net. The FPN model underperformed as the evaluation metrics were the least among the rest of the models. Overall, the performance of all four pixel-based models was very similar.

The PR and ROC curves for the pixel-based models were plotted (Supplementary Fig. 3). The mean PR AUC and mean ROC AUC scores followed a similar trend as the evaluation metrics (Supplementary Table 3). The U-Net, MA-Net, and DeepLabv3 + models showed the same PR AUC and PRC AUC values of 0.88/0.94, slightly higher than that of the FPN model (0.86/0.94). Hence, based on the evaluation metrics, we concluded that, for patch inference, the U-Net, MA-Net, and DeepLabv3 + models were the best performing models.

#### Whole image inference

In whole image inference, test images were not divided into patches. Instead, full images were passed through the trained models and the predictions were obtained. Supplementary Table 4 shows the evaluation metrics for all the semantic segmentation-based models using whole image inference. We found that the U-Net model outperformed all the other models across all different evaluation metrics except for recall. The next best performing model was the MA-Net that was followed by FPN. The DeepLabv3 + provided the least results as their evaluation metrics were minimal when compared to all other models except for specificity. We observed that the U-Net/MA-Net had mean Jaccard of 0.79/0.78 and mean Dice coefficient of 0.88/0.87, while the FPN/DeepLabv3 + had mean Jaccard of 0.77/0.76 and mean Dice coefficient of 0.86/0.86.

We also plotted the PR curves (Fig. 5E-H) and ROC curves (Fig. 6E-H) for all the models in the whole image inference. The PR AUC and ROC AUC followed a similar trend as the evaluation metrics (Supplementary Table 4). The U-Net model resulted in higher PR AUC/ROC AUC values of 0.92/0.96 in comparison with other models. U-Net was followed by MA-Net, DeepLabv3 + and FPN. Hence, based on the evaluation metrics, we concluded that, for whole image-based inference, the U-Net was the best performing model. For both patch-based inference and whole-image-based inference, U-Net consistently outperformed other models.

### Comparison of patch-based and pixel-based approach

**Fig. 7:**
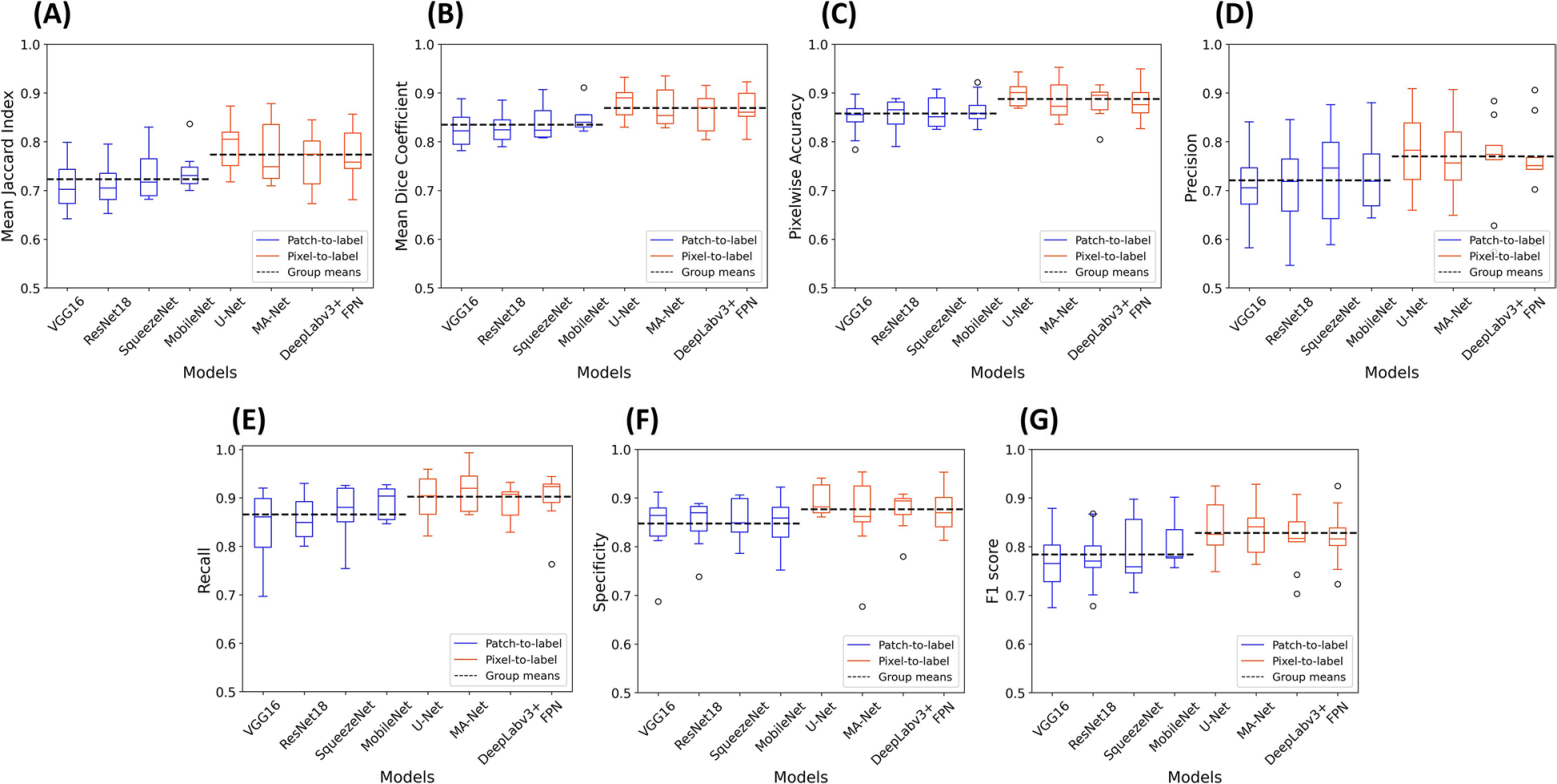
Comparison of patch-based (blue) and pixel-based (orange) models with whole image-based inference in Mean Jaccard Index (**A**), Mean Dice Coefficient (**B**), Pixelwise Accuracy (**C**), Precision (**D**), Recall (**E**), Specificity (**F**), and F1 Score (**G**). The models were evaluated by ninefold cross-validation and the seven TUR images in each fold were used to calculate the evaluation metrics. For patch-based or pixel-based models respectively, the group means are indicated by black dashed lines

To compare the performance of four patch-based and four pixel-based models using whole image-based inference as groups, we made plots for different evaluation metrics, including mean Jaccard index (Fig. 7A), mean Dice coefficient (Fig. 7B), pixelwise accuracy (Fig. 7C), precision (Fig. 7D), recall (Fig. 7E), specificity (Fig. 7F), and F1 score (Fig. 7G). Clearly, for each evaluation metric, the average value of pixel-based approach as a group was higher than that of patch-based approach, which was consistent with the findings in the previous study [33]. Compared to the patch-based models, the outperformance of the pixel-based models was not surprising because this approach was tailor-made for the semantic segmentation task. However, the patch-based approach still provided stiff competition to the pixel-based approach in all evaluation metrics. Supplementary Figure 4 shows the performance comparison results for the four patch-based models and four pixel-based models using patch-based inference.

Notably, pixel-based models, on average, had a higher number of parameters than patch-based models. Particularly, pixel-based models had 12 to 21 million parameters whereas patch-based models (except VGG16) had 0.74 to 11 million parameters. As a result, the training time for pixel-based models (12–17 h) was longer than that for patch-based models (< 5 h) (Table 1).

### Visualization of segmentation results

**Fig. 8:**
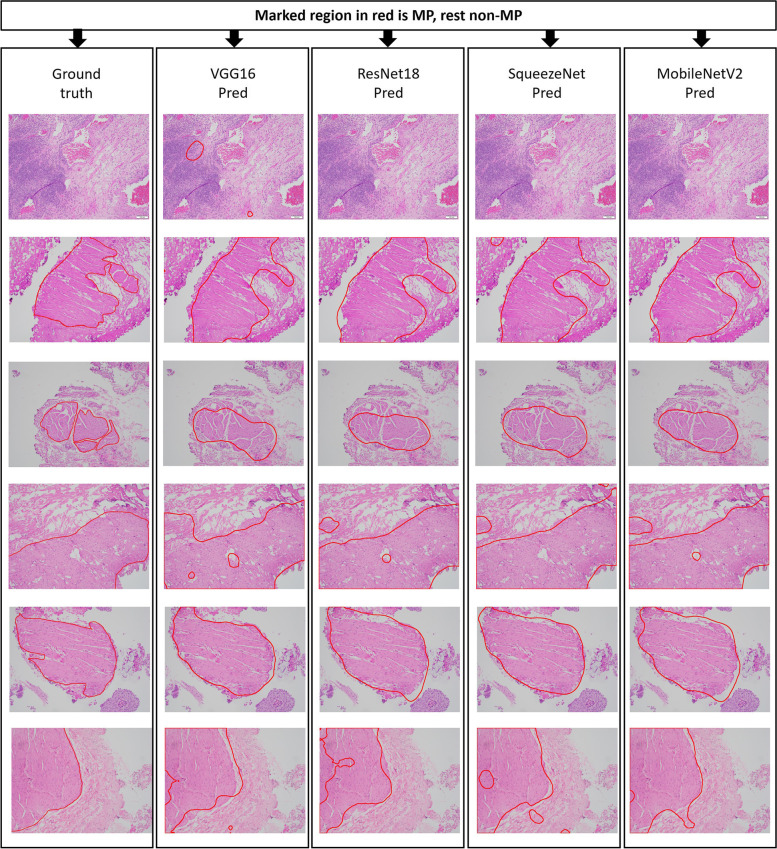
Segmentation results of test TUR images using patch-based models (VGG16, ResNet18, SqueezeNet, and MobileNetV2). The first column represents the ground truth marked by the expert pathologists. The subsequent columns indicate the segmentation results from corresponding models

**Fig. 9:**
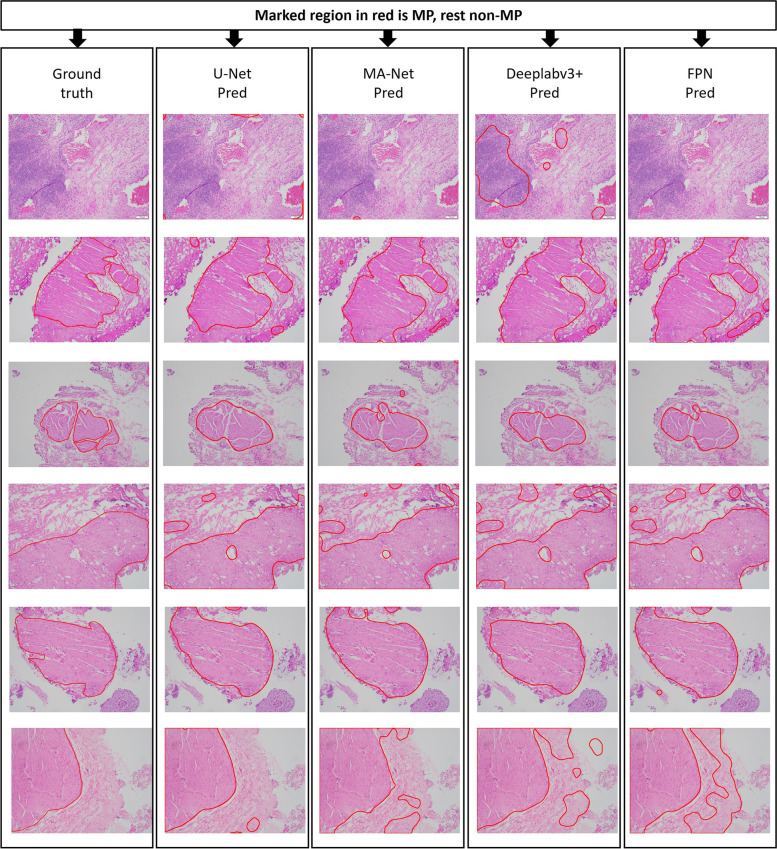
Segmentation results of test TUR images using pixel-based models (U-Net, MA-Net, DeepLabv3 + , and FPN) with whole image-based inference. The first column represents the ground truth marked by the expert pathologists. The subsequent columns indicate the segmentation results from corresponding models

To further compare the performance of proposed models, we visualized the segmentation results of several test TUR images obtained from the patch-based models (Fig. 8) and the pixel-based models with whole image inference (Fig. 9). Supplementary Figure 5 shows the results for the pixel-based models with patch inference. In all cases, the predicted MP regions from the models were compared with the ground truths in which MP regions were marked by pathologists (Column 1 in Figs. 8 and 9). Overall, all models provided predictions similar to the ground truths. In some cases, MobileNetV2 gave a better prediction than other patch-based models (for example last row in Fig. 8), consistent with our data showing that MobileNetV2 outperformed other models in the evaluation metrics (Supplementary Table 2). Similarly, in some cases, U-Net presented a better prediction than other pixel-based models (for example last row in Fig. 9), consistent with our data showing that U-Net was the best performer (Supplementary Table 4). After comparing the model performances in both the approaches, we were curious to see model MP predictions for special case input images such as images containing both MP and MM muscle fibers. Thus, these images were passed through the best models in both the approaches, i.e., MobileNetV2 for patch-based approach and U-Net for pixel-based approach (both patch-based and whole image-based inferences), and the visualizations are represented in Supplementary Figure 6. Thus, MobileNetV2 and U-Net (for both patch-based and whole image-based inferences) correctly predicted MP regions and non-MP regions even when the image contains both MP and MM muscle fibers.

## Discussion

We have performed the first computational analysis of smooth muscle in H&E images using two groups of DL model approaches – patch-based approach and pixel-based approach and compared their performances numerically as well as visually. We used four representative models from each group and evaluated them by seven metrics. Both groups of the models successfully identified and segmented MP regions in histopathological images obtained from H&E-stained bladder specimens, with pixelwise accuracy ranging from 0.85–0.87 (patch-based models) to 0.88–0.90 (pixel-based models). We further showed that MobileNetV2 and U-Net outperform their peers in the patch-based and pixel-based model groups, respectively. We found that the pixel-based models, on average, have better performance in all evaluation metrics than the patch-based models. However, the pixel-based models have more training parameters, which leads to longer training time and requires more computational resources. Our data suggested that both types of approaches are able to help pathologists as a computer-aided diagnostic system. That is, given an image of the tissue region that appears unclear to a pathologist, our trained models can provide a convenient way to differentiate MP from non-MP tissues. This information will help the pathologist make an accurate diagnosis.

Our work on smooth muscle segmentation is different from the published work on skeletal muscle segmentation [35–41]. This is because smooth muscle is morphologically distinct from skeletal muscle. First, smooth muscle is non-striated whereas skeletal muscle has transverse streaks. Second, each smooth muscle cell has one central nucleus whereas a skeletal muscle cell often has multiple nuclei. These microscopically distinctive features suggest that a new system is required for smooth muscle segmentation. Our work represents the first step towards this goal, laying the groundwork for further development of computational models to characterize smooth muscles.

## Conclusions

In this study, we made a comprehensive comparison between two types of classification models – patch-based and pixel-based approaches, for segmenting the MP and non-MP (including MM) regions in bladder tissues obtained by TUR or RC. Various metrics were used for model performance evaluation. We found that the pixel-based models, in general, outperformed the patch-based models in almost all metrics. In particular, U-Net was the best pixel-based model whereas MobileNetV2 was the best patch-based model. Our work provides the first computer-aided diagnostic system that reliably distinguishes between MP (MIBC) vs. non-MP (NMIBC) invasion in surgical specimens, which has a significant clinical impact on pathological staging of bladder cancer and decision making in the patient management. This system can be further improved by utilizing traditional ML techniques such as Random Forest, Support Vector Machine or K-Nearest Neighbor on top of the DL architecture to classify MP vs. non-MP, given the relatively small sample size. In addition, instead of using the deep networks pretrained by ImageNet, other networks such as KimiaNet may need to be tested, which is a DenseNet121 architecture Imagenet-pretrained CNN model fine-tuned on over 250,000 histopathology images.

## Consent for publication

Not applicable.

## Supplementary Information

**Additional file 1: Supplementary Figure 1.** Illustration of steps involved in post processing the model output, high resolution heatmap image. The heatmap is passed through adaptive thresholding, median and average filtering, and Otsu thresholding to obtain smooth noise-free output-images.** Supplementary Figure 2.** Illustration of generating labels (ground truth) from pathologists’ annotated images using MATLAB® image labeler tool. The ground truth is a binary image representing the MP region as white pixels and the non-MP region as black pixels.** Supplementary Figure 3.** PR curves (left) and ROC curves (right) for classification of MP and non-MP regions by U-Net (A), MA-Net (B), DeepLabv3+ (C) and FPN (D) models. The models were trained and tested using 9-fold cross-validation. Seven TUR images were evaluated for each fold and corresponding PR-AUC and ROC-AUC values are indicated in the caption. The mean PR curve (blue) and standard deviation (grey shaded region) are provided for the models.** Supplementary Figure 4.** Comparison of patch-based (blue) and pixel-based (orange) models with patch-based inference in Mean Jaccard Index (A), Mean Dice Coefficient (B), Pixelwise Accuracy (C), Precision (D), Recall (E), Specificity (F), and F1 Score (G). The models were evaluated by 9-fold cross-validation and the seven TUR images in each fold were used to calculate the evaluation metrics. For patch-based or pixel-based models, the group means are indicated by dashed lines.** Supplementary Figure 5.** Segmentation results of test TUR images using pixel-based models (U-Net, MA-Net, DeepLabv3+, and FPN) with patch-based inference. The first column represents the ground truth marked by the expert pathologists. The subsequent columns indicate the segmentation results from corresponding models.** Supplementary Figure 6.** Segmentation results of special case images using best models in both the approaches, i.e., MobileNetV2 for patch-based approach and U-Net for pixel-based approach (both patch-based and whole image-based inferences). The first column represents the ground truth marked by the expert pathologists. The subsequent columns indicate the segmentation results from corresponding models.** Supplementary Table 1.** Summary of machine specifications.** Supplementary Table 2.** Performance of patch-based models (best performers shown in bold).** Supplementary Table 3.** Performance of pixel-based models (patch-based inference with the best performers shown in bold).** Supplementary Table 4.** Performance of pixel-based models (whole image inference with the best performers shown in bold).

## Data Availability

Histopathological images and corresponding ground truth datasets from the current study are not publicly available due to large file size but are available from the corresponding authors on reasonable requests. Processing pipeline is publicly available at https://github.com/rit-cui-lab/Deep-learning-for-histopathological-segmentation-of-smooth-muscle-in-the-urinary-bladder. No identifying/ confidential patient data was collected.

## Abbreviations

MM: Muscularis mucosa
MP: Muscularis propria
DL: Deep learning
NMIBC: Non-muscle-invasive bladder cancer
MIBC: Muscle-invasive bladder cancer
RC: Radical cystectomy
TUR: Transurethral resection
H&E: Hematoxylin & eosin
ML: Machine learning
WSI: Whole-slide-image
CNN: Convolutional neural network
TP: True positive
FP: False positive
TN: True negative
FN: False negative
SGD: Stochastic gradient descent
URMC: University of Rochester Medical Center
PR: Precision-Recall
ROC: Receiver Operator Characteristic
AUC: Area under the curve

## References

[1] Dixon J, Gosling J (1983). Histology and fine structure of the muscularis mucosae of the human urinary bladder. J Anat.

[2] Sung H (2021). “Global cancer statistics 2020: GLOBOCAN estimates of incidence and mortality worldwide for 36 cancers in 185 countries”. CA Cancer J= Clin.

[3] Paner GP, Ro JY, Wojcik EM, Venkataraman G, Datta MW, Amin MB (2007). "Further characterization of the muscle layers and lamina propria of the urinary bladder by systematic histologic mapping: implications for pathologic staging of invasive urothelial carcinoma," (in eng). Am J Surg Pathol.

[4] Miyamoto H, Epstein JI (2010). "Transurethral resection specimens of the bladder: outcome of invasive urothelial cancer involving muscle bundles indeterminate between muscularis mucosae and muscularis propria," (in eng). Urology.

[5] Miyamoto H, Sharma RB, Illei PB, Epstein JI (2010). "Pitfalls in the use of smoothelin to identify muscularis propria invasion by urothelial carcinoma," (in eng). Am J Surg Pathol.

[6] Elkady N, Abdou AG, Kandil M, Ghanem N (2017). "Diagnostic value of smoothelin and vimentin in differentiating muscularis propria from muscularis mucosa of bladder carcinoma," (in eng). Int J Biol Markers.

[7] El-Baz A, Gimel'farb G, Suzuki K (2017). "Machine learning applications in medical image analysis," (in eng). Comput Math Methods Med.

[8] Shen D, Wu G, Suk H-I (2017). Deep learning in medical image analysis. Annu Rev Biomed Eng.

[9] Srinidhi CL, Ciga O, Martel AL (2021). "Deep neural network models for computational histopathology: A survey," (in eng). Med Image Anal.

[10] Cireşan DC, Giusti A, Gambardella LM, Schmidhuber J (2013). "Mitosis detection in breast cancer histology images with deep neural networks," (in eng). Med Image Comput Comput Assist Interv.

[11] Cruz-Roa A (2014). "Automatic detection of invasive ductal carcinoma in whole slide images with convolutional neural networks," in Medical Imaging 2014. Digit Pathol.

[12] Long J, Shelhamer E, and Darrell T. Fully convolutional networks for semantic segmentation. Proceedings of the IEEE conference on computer vision and pattern recognition. 2015. 10.48550/arXiv.1411.4038.

[13] Chen H, Qi X, Yu L, Dou Q, Qin J, Heng PA (2017). "DCAN: Deep contour-aware networks for object instance segmentation from histology images," (in eng). Med Image Anal.

[14] Xu Y, Zhu JY, Chang EI, Lai M, Tu Z (2014). "Weakly supervised histopathology cancer image segmentation and classification," (in eng). Med Image Anal.

[15] Kingma DP, Welling M. Auto-Encoding Variational Bayes. arXiv. 2013. 10.48550/arXiv.1312.6114.

[16] Higgins I, Matthey L, Pal A, Burgess C, Glorot X, Botvinick M, Mohamed S, Lerchner A. Beta-VAE: Learning basic visual concepts with a constrained variational framework. Proceedings of ICLR. 2017. https://openreview.net/pdf?id=Sy2fzU9gl.

[17] Chen X, Duan Y, Houthooft R, Schulman J, Sutskever I, Abbeel P. Infogan: Interpretable representation learning by information maximizing generative adversarial nets. Advances in neural information processing systems 2016;29. 10.48550/arXiv.1606.03657.

[18] Chang H, Han J, Zhong C, Snijders AM, Mao JH (2018). "Unsupervised transfer learning via multi-scale convolutional sparse coding for biomedical applications," (in eng). IEEE Trans Pattern Anal Mach Intell.

[19] Wang D, Khosla A, Gargeya R, Irshad H, Beck AH. Deep learning for identifying metastatic breast cancer. arXiv. 2016. 10.48550/arXiv.1606.05718.

[20] Liu Y, et al. Detecting cancer metastases on gigapixel pathology images. arXiv. 2017. 10.48550/arXiv.1703.02442.

[21] Arvaniti E (2018). "Automated Gleason grading of prostate cancer tissue microarrays via deep learning," (in eng). Sci Rep.

[22] Simonyan K, Zisserman A. Very deep convolutional networks for large-scale image recognition. arXiv. 2014. 10.48550/arXiv.1409.1556.

[23] He K, Zhang X, Ren S, Sun J. Deep residual learning for image recognition. Proceedings of the IEEE conference on computer vision and pattern recognition. 2016. 10.48550/arXiv.1512.03385.

[24] Iandola FN, Han S, Moskewicz MW, Ashraf K, Dally WJ, Keutzer K. SqueezeNet: AlexNet-level accuracy with 50x fewer parameters and <0.5 MB model size. arXiv. 2016. 10.48550/arXiv.1602.07360.

[25] Sandler M, Howard A, Zhu M, Zhmoginov A, Chen L-C. Mobilenetv2: Inverted residuals and linear bottlenecks. Proceedings of the IEEE conference on computer vision and pattern recognition. 2018. 10.48550/arXiv.1801.04381.

[26] Howard A, et al. Searching for mobilenetv3. Proceedings of the IEEE/CVF International Conference on Computer Vision. 2019. 10.48550/arXiv.1905.02244.

[27] Le H (2020). Utilizing automated breast cancer detection to identify spatial distributions of tumor-infiltrating lymphocytes in invasive breast Cancer. Am J Pathol.

[28] Litjens G (2016). "Deep learning as a tool for increased accuracy and efficiency of histopathological diagnosis," (in eng). Sci Rep.

[29] Sekou TB, Hidane M, Olivier J, Cardot H. From Patch to Image Segmentation using Fully Convolutional Networks--Application to Retinal Images. arXiv. 2019. 10.48550/arXiv.1904.03892.

[30] Bulten W (2019). Epithelium segmentation using deep learning in H&E-stained prostate specimens with immunohistochemistry as reference standard. Sci Rep.

[31] Taghanaki SA, Abhishek K, Cohen JP, Cohen-Adad J, Hamarneh G (2021). Deep semantic segmentation of natural and medical images: a review. Artif Intell Rev.

[32] Ronneberger O, Fischer P, Brox T (2015). U-net: Convolutional networks for biomedical image segmentation. International Conference on Medical image computing and computer-assisted intervention.

[33] Jiménez G, Racoceanu D (2019). "Deep learning for semantic segmentation vs. classification in computational pathology: application to mitosis analysis in breast cancer grading," (in eng). Front Bioeng Biotechnol.

[34] Fan T, Wang G, Li Y, Wang H (2020). MA-Net: a multi-scale attention network for liver and tumor segmentation. IEEE Access.

[35] Chen L-C, Zhu Y, Papandreou G, Schroff F, Adam H. Encoder-decoder with atrous separable convolution for semantic image segmentation," Proceedings of the European conference on computer vision (ECCV). 2018. 10.48550/arXiv.1802.02611.

[36] Lin T-Y, Dollár P, Girshick R, He K, Hariharan B, Belongie S. Feature pyramid networks for object detection. Proceedings of the IEEE conference on computer vision and pattern recognition. 2017. 10.48550/arXiv.1612.03144.

[37] Paszke A. et al. Pytorch: An imperative style, high-performance deep learning library. arXiv. 2019. 10.48550/arXiv.1912.01703.

[38] Harris CR (2020). "Array programming with NumPy," (in eng). Nature.

[39] McKinney W (2010). Data structures for statistical computing in python. Proc 9th Python Sci Conference.

[40] Deng J, Dong W, Socher R, Li L-J, Li K, Li F-F. Imagenet: A large-scale hierarchical image database. IEEE conference on computer vision and pattern recognition. 2009. https://ieeexplore.ieee.org/document/5206848.

[41] Bozinovski S. Reminder of the first paper on transfer learning in neural networks, 1976. Informatica. 2020. https://www.informatica.si/index.php/informatica/article/view/2828/1433.

[42] Kim YG (2020). "Effectiveness of transfer learning for enhancing tumor classification with a convolutional neural network on frozen sections" (in eng). Sci Rep.

[43] Youden WJ (1950). "Index for rating diagnostic tests," (in eng). Cancer.

[44] Bengio Y (2012). "Practical recommendations for gradient-based training of deep architectures," in Neural networks: Tricks of the trade.

[45] Rother C, Kolmogorov V, Blake A (2004). " GrabCut" interactive foreground extraction using iterated graph cuts. ACM transact Graph (TOG).

[46] Reinhard E, Ashikhmin M, Gooch B, Shirley P. Color Transfer between Images. IEEE Comput. Graph. Appl. 2001. https://ieeexplore.ieee.org/abstract/document/946629.

[47] Cohen MP, Lovric M (2011). "Stratified Sampling,". International encyclopedia of statistical science.

