# Supplementary material for: Deep learning for histopathological segmentation of smooth muscle in the urinary bladder
Source: BMC Med Inform Decis Mak. 2023 Jul 15;23:122. doi: 10.1186/s12911-023-02222-3 (PMC10349433; doi:10.1186/s12911-023-02222-3)
Supplement: Supplementary file 1 — Additional file 1: Supplementary Figure 1. Illustration of steps involved in post processing the model output, high resolution heatmap image. The heatmap is passed through adaptive thresholding, median and average filtering, and Otsu thresholding to obtain smooth noise-free output-images. Supplementary Figure 2. Illustration of generating labels (ground truth) from pathologists’ annotated images using MATLAB® image labeler tool. The ground truth is a binary image representing the MP region as white pixels and the non-MP region as black pixels. Supplementary Figure 3. PR curves (left) and ROC curves (right) for classification of MP and non-MP regions by U-Net (A), MA-Net (B), DeepLabv3+ (C) and FPN (D) models. The models were trained and tested using 9-fold cross-validation. Seven TUR images were evaluated for each fold and corresponding PR-AUC and ROC-AUC values are indicated in the caption. The mean PR curve (blue) and standard deviation (grey shaded region) are provided for the models. Supplementary Figure 4. Comparison of patch-based (blue) and pixel-based (orange) models with patch-based inference in Mean Jaccard Index (A), Mean Dice Coefficient (B), Pixelwise Accuracy (C), Precision (D), Recall (E), Specificity (F), and F1 Score (G). The models were evaluated by 9-fold cross-validation and the seven TUR images in each fold were used to calculate the evaluation metrics. For patch-based or pixel-based models, the group means are indicated by dashed lines. Supplementary Figure 5. Segmentation results of test TUR images using pixel-based models (U-Net, MA-Net, DeepLabv3+, and FPN) with patch-based inference. The first column represents the ground truth marked by the expert pathologists. The subsequent columns indicate the segmentation results from corresponding models. Supplementary Figure 6. Segmentation results of special case images using best models in both the approaches, i.e., MobileNetV2 for patch-based approach and U-Net for pixel-based approach (both patch [file 12911_2023_2222_MOESM1_ESM.docx]

***Supplementary Material***

# Supplementary Figures

**Supplementary Figure 1.** Illustration of steps involved in post processing the model output, high resolution heatmap image. The heatmap is passed through adaptive thresholding, median and average filtering, and Otsu thresholding to obtain smooth noise-free output-images.
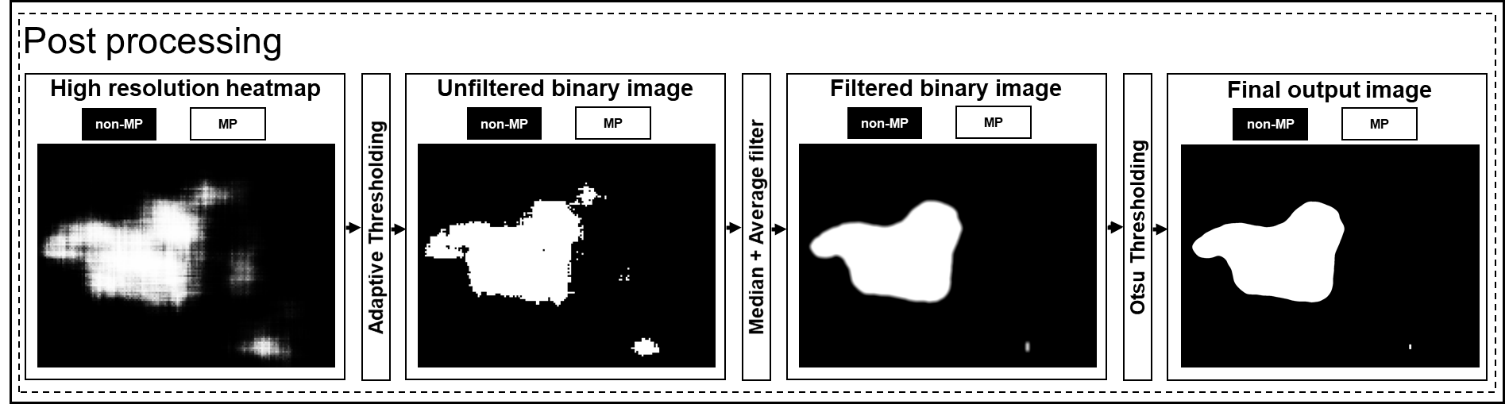


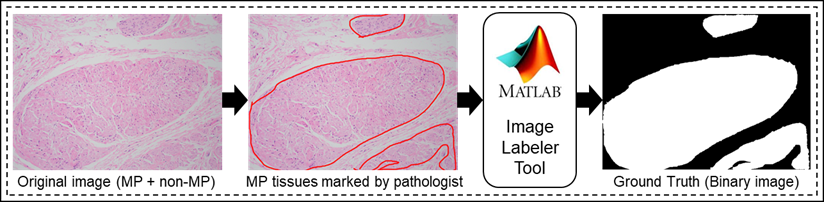
**Supplementary Figure 2** **.** Illustration of generating labels (ground truth) from pathologists’ annotated images using MATLAB® image labeler tool. The ground truth is a binary image representing the MP region as white pixels and the non-MP region as black pixels.


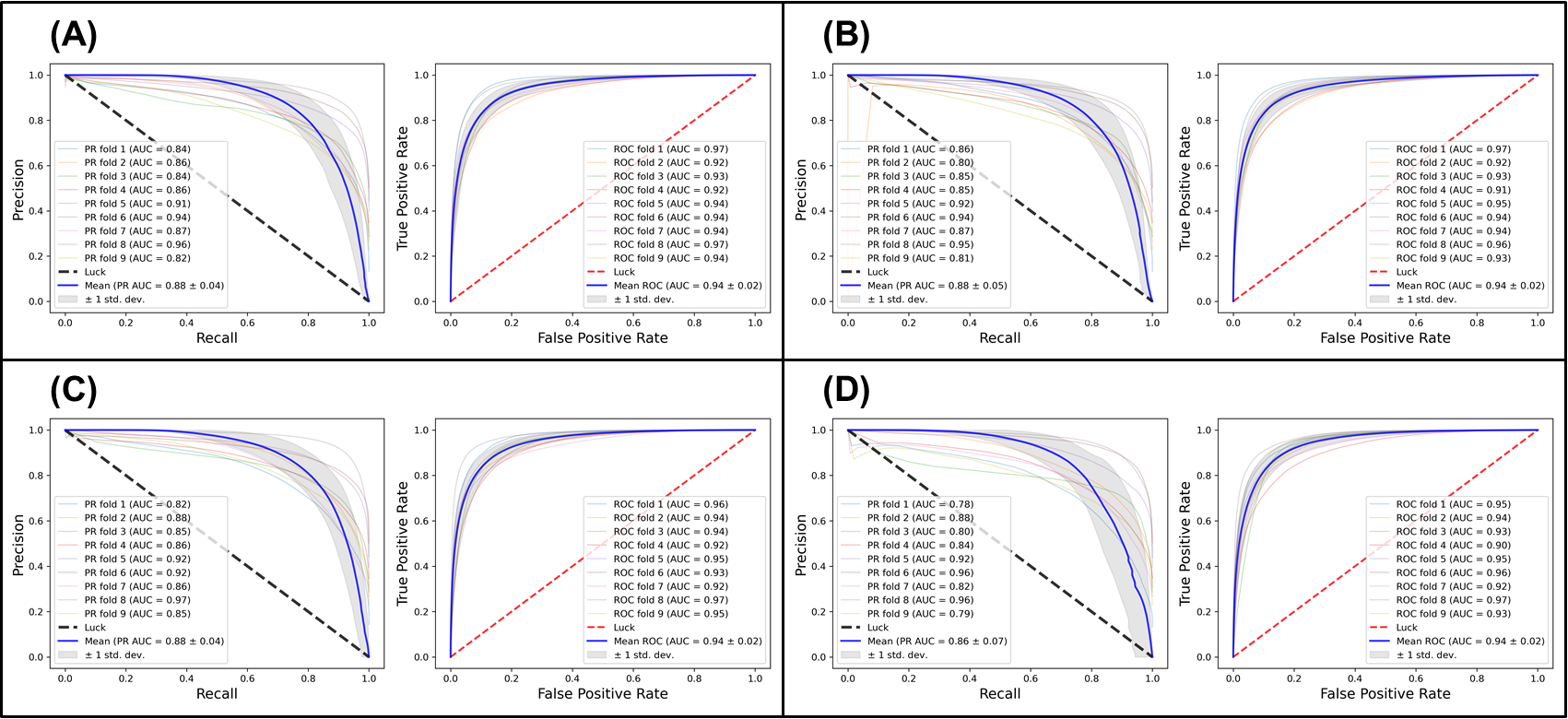


**Supplementary Figure 3** **.** PR curves (left) and ROC curves (right) for classification of MP and non-MP regions by U-Net (A), MA-Net (B), DeepLabv3+ (C) and FPN (D) models. The models were trained and tested using 9-fold cross-validation. Seven TUR images were evaluated for each fold and corresponding PR-AUC and ROC-AUC values are indicated in the caption. The mean PR curve (blue) and standard deviation (grey shaded region) are provided for the models.


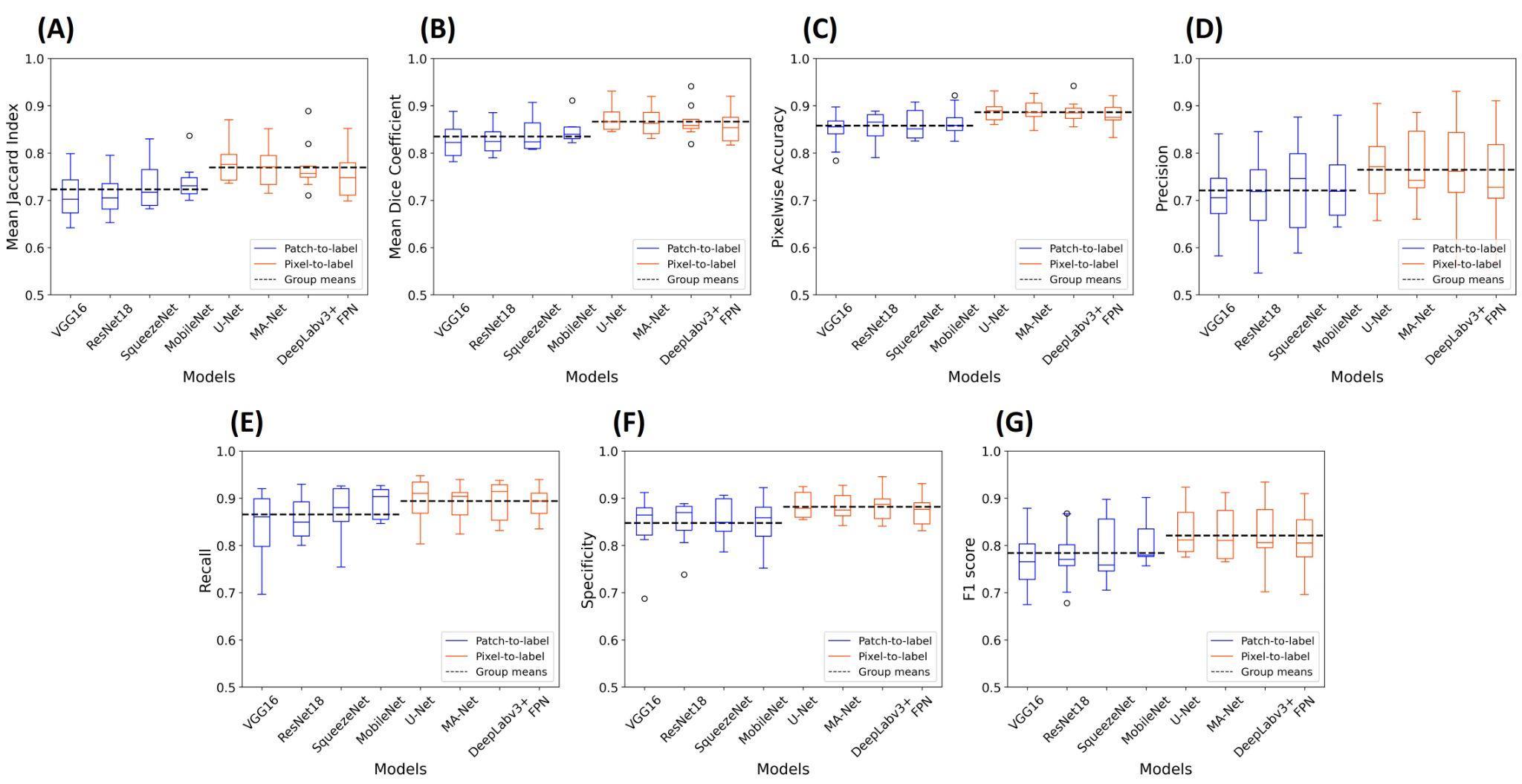


**Supplementary Figure 4.** Comparison of patch-based (blue) and pixel-based (orange) models with patch-based inference in Mean Jaccard Index (A), Mean Dice Coefficient (B), Pixelwise Accuracy (C), Precision (D), Recall (E), Specificity (F), and F1 Score (G). The models were evaluated by 9-fold cross-validation and the seven TUR images in each fold were used to calculate the evaluation metrics. For patch-based or pixel-based models, the group means are indicated by dashed lines.


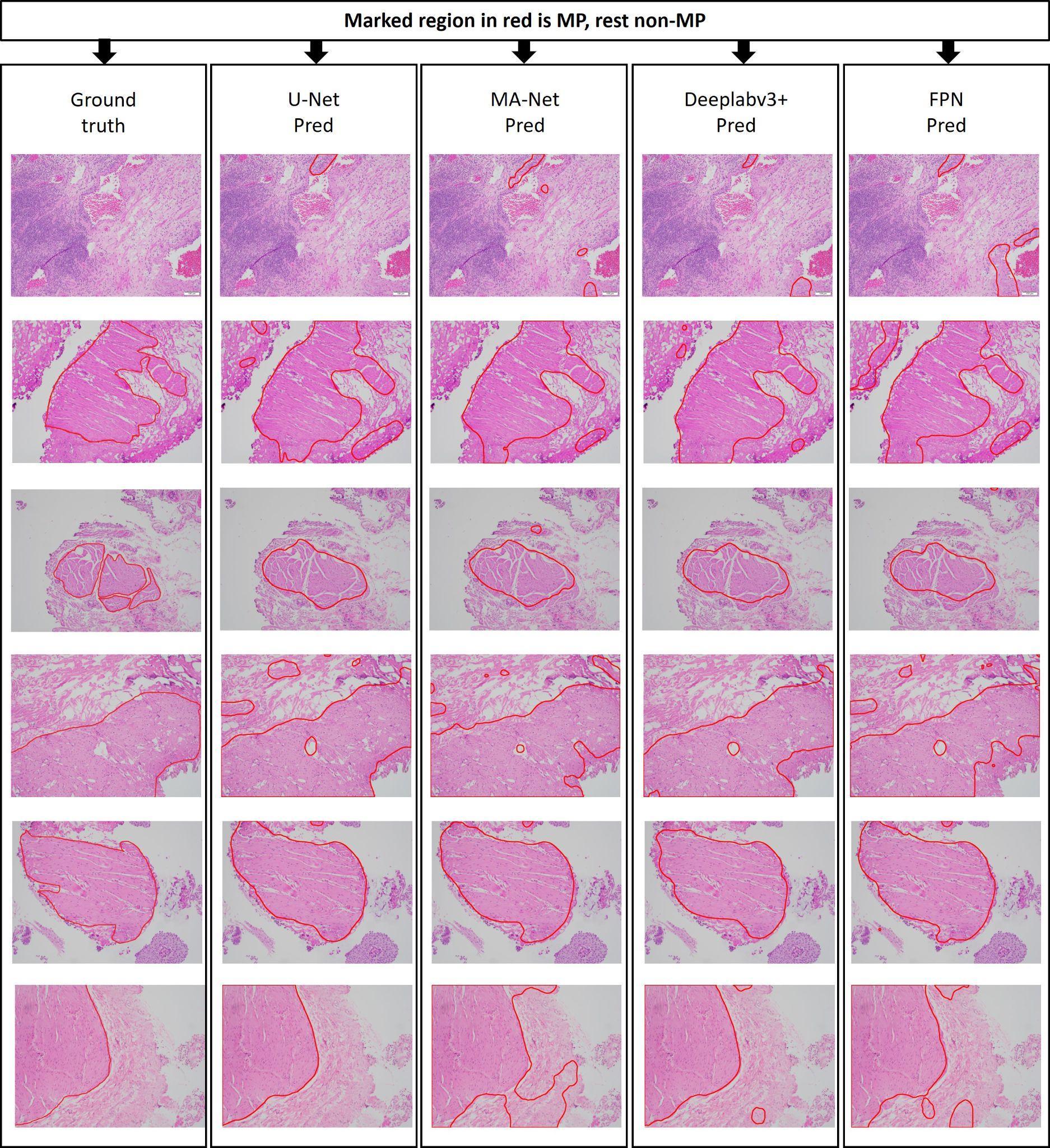


**Supplementary Figure 5** **.** Segmentation results of test TUR images using pixel-based models (U-Net, MA-Net, DeepLabv3+, and FPN) with patch-based inference. The first column represents the ground truth marked by the expert pathologists. The subsequent columns indicate the segmentation results from corresponding models.


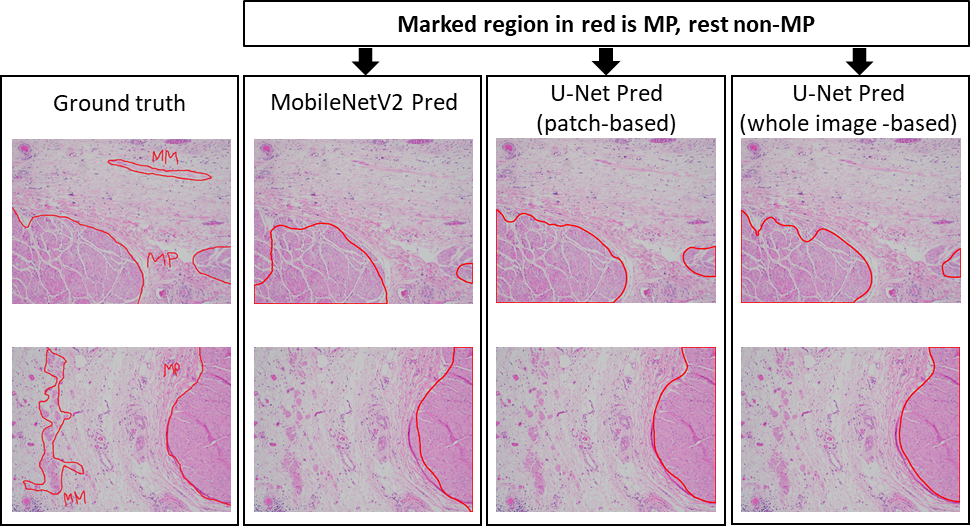


**Supplementary Figure 6.** Segmentation results of special case images using best models in both the approaches, i.e., MobileNetV2 for patch-based approach and U-Net for pixel-based approach (both patch-based and whole image-based inferences). The first column represents the ground truth marked by the expert pathologists. The subsequent columns indicate the segmentation results from corresponding models.

# Supplementary Tables

**Supplementary Table 1.** Summary of machine specifications

| **Hardware** | **Software** |  |
| --- | --- | --- |
|  |  |  |
| Random Access Memory (RAM) 16GB  Processor Intel(R) Core(TM) i7-10750H CPU @ 2.6GHz, 2592 Mhz, 6 Core(s), 12 Logical Processor(s)  Graphics NVIDIA GeForce GTX 1660 Ti  Operating system Windows 10 Home 64-bit (10.0, Build 19042) | Integrated development environment (IDE) Anaconda Spyder (Python 3.8)  Libraries PyTorch, Pandas, NumPy, OpenCV, Scikit-learn, Matplotlib  Ground truth preparation MATLabR2020b |  |
|  |  |  |
|  |  |  |
|  |  |  |
|  |  |  |
|  |  |  |
|  |  |  |
|  |  |  |
|  |  |  |
|  |  |  |
|  |  |  |
|  |  |  |

**Supplementary Table 2.** Performance of patch-based models (best performers shown in bold)

| **Evaluation metrics** | **VGG16** | **ResNet18** | **SqueezeNet** | **MobileNetV2** |
| --- | --- | --- | --- | --- |
|  | **(mean ± std. dev.)** | **(mean ± std. dev.)** | **(mean ± std. dev.)** | **(mean ± std. dev.)** |
| Mean Jaccard Index | 0.71 ± 0.05 | 0.72 ± 0.05 | 0.73 ± 0.05 | **0.74 ± 0.04** |
| Mean Dice co-efficient | 0.82 ± 0.03 | 0.83 ± 0.03 | 0.84 ± 0.03 | **0.85 ± 0.03** |
| Pixelwise Accuracy | 0.85 ± 0.03 | 0.85 ± 0.03 | 0.86 ± 0.03 | **0.87 ± 0.03** |
| Precision | 0.71 ± 0.07 | 0.71 ± 0.09 | 0.73 ± 0.09 | **0.73 ± 0.07** |
| Recall | 0.84 ± 0.07 | 0.86 ± 0.04 | 0.87 ± 0.05 | **0.89 ± 0.03** |
| Specificity | 0.84 ± 0.06 | **0.86 ± 0.05** | 0.85 ± 0.04 | 0.85 ± 0.05 |
| F1 Score | 0.77 ± 0.06 | 0.78 ± 0.06 | 0.79 ± 0.06 | **0.80 ± 0.04** |

**Supplementary Table 3.** Performance of pixel-based models (patch-based inference with the best performers shown in bold)

| **Evaluation metrics** | **U-Net (mean ± std. dev.)** | **MA-Net (mean ± std. dev.)** | **DeepLabv3+ (mean ± std. dev.)** | **FPN (mean ± std. dev.)** |
| --- | --- | --- | --- | --- |
| Mean Jaccard Index | **0.78 ± 0.04** | 0.77 ± 0.04 | 0.77 ± 0.05 | 0.76 ± 0.05 |
| Mean Dice co-efficient | **0.87 ± 0.03** | **0.87 ± 0.03** | **0.87 ± 0.03** | 0.86 ± 0.03 |
| Pixelwise Accuracy | **0.89 ± 0.02** | 0.89 ± 0.03 | **0.89 ± 0.02** | **0.89 ± 0.02** |
| Precision | **0.77 ± 0.08** | **0.77 ± 0.08** | 0.77 ± 0.10 | 0.75 ± 0.10 |
| Recall | **0.90 ± 0.04** | 0.89 ± 0.03 | **0.90 ± 0.04** | 0.89 ± 0.03 |
| Specificity | **0.88 ± 0.03** | **0.88 ± 0.03** | **0.88 ± 0.03** | 0.87 ± 0.03 |
| F1 Score | **0.83 ± 0.05** | 0.82 ± 0.05 | 0.82 ± 0.06 | 0.81 ± 0.07 |

**Supplementary Table 4.** Performance of pixel-based models (whole image inference with the best performers shown in bold)

| **Evaluation metrics** | **U-Net** | **MA-Net** | **DeepLabv3+** | **FPN** |
| --- | --- | --- | --- | --- |
|  | **(mean ± std. dev.)** | **(mean ± std. dev.)** | **(mean ± std. dev.)** | **(mean ± std. dev.)** |
| Mean Jaccard Index | **0.79 ± 0.05** | 0.78 ± 0.06 | 0.76 ± 0.05 | 0.77 ± 0.06 |
| Mean Dice co-efficient | **0.88 ± 0.03** | 0.87 ± 0.04 | 0.86 ± 0.04 | 0.86 ± 0.04 |
| Pixelwise Accuracy | **0.90 ± 0.03** | 0.89 ± 0.04 | 0.88 ± 0.03 | 0.88 ± 0.04 |
| Precision | **0.79 ± 0.08** | 0.77 ± 0.08 | 0.76 ± 0.09 | 0.76 ± 0.08 |
| Recall | 0.90 ± 0.04 | **0.92 ± 0.04** | 0.89 ± 0.03 | 0.90 ± 0.05 |
| Specificity | **0.89 ± 0.03** | 0.87 ± 0.08 | 0.88 ± 0.04 | 0.87 ± 0.04 |
| F1 Score | **0.84 ± 0.05** | 0.83 ± 0.05 | 0.82 ± 0.06 | 0.82 ± 0.06 |
